# New Insights into Antibacterial and Antifungal Properties, Cytotoxicity and Aquatic Ecotoxicity of Flame Retardant PA6/DOPO-Derivative Nanocomposite Textile Fibers

**DOI:** 10.3390/polym13060905

**Published:** 2021-03-15

**Authors:** Jelena Vasiljević, Danaja Štular, Gabriela Kalčíková, Janja Zajc, Matic Šobak, Andrej Demšar, Brigita Tomšič, Barbara Simončič, Marija Čolović, Vid Simon Šelih, Ivan Jerman

**Affiliations:** 1 Faculty of Natural Sciences and Engineering, University of Ljubljana, Aškerčeva 12, 1000 Ljubljana, Slovenia; andrej.demsar@ntf.uni-lj.si (A.D.); brigita.tomsic@ntf.uni-lj.si (B.T.); barbara.simoncic@ntf.uni-lj.si (B.S.); 2 National Institute of Chemistry, Hajdrihova 19, 1000 Ljubljana, Slovenia; danaja.stular@ki.si (D.Š.); matic.sobak@ki.si (M.Š.); marija.colovic@ki.si (M.Č.); vid.selih@ki.si (V.S.Š.); ivan.jerman@ki.si (I.J.); 3 Faculty of Chemistry and Chemical Technology, University of Ljubljana, Večna pot 113, 1000 Ljubljana, Slovenia; gabriela.kalcikova@fkkt.uni-lj.si; 4 Agricultural Institute of Slovenia, Hacquetova 17, 1000 Ljubljana, Slovenia; janja.zajc@kis.si

**Keywords:** polyamide 6, textile filament, DOPO derivative, antibacterial activity, antifungal activity, ecotoxicity, cytotoxicity

## Abstract

The aim of this study was to evaluate the antibacterial and antifungal activity, cytotoxicity, leaching, and ecotoxicity of novel flame retardant polyamide 6 (PA6) textile fibers developed by our research group. The textile fibers were produced by the incorporation of flame-retardant bridged 9,10-dihydro-9-oxa-10-phosphaphenanthrene-10-oxide (DOPO) derivative (PHED) in the PA6 matrix during the in situ polymerization process at concentrations equal to 10 and 15 wt% (PA6/10PHED and PA6/15PHED, respectively). Whilst the nanodispersed PHED provided highly efficient flame retardancy, its biological activity led to excellent antibacterial activity against *Escherichia coli* and *Staphylococcus aureus*, as well as excellent antifungal activity against *Aspergillus niger* and *Candida albicans*. The results confirmed leaching of the PHED, but the tested leachates did not cause any measurable toxic effect to the duckweed Lemna minor. The in vitro cytotoxicity of the leached PHED from the PA6/15PHED sample was confirmed for human cells from adipose tissue in direct and prolonged contact. The targeted biological activity of the organophosphinate flame retardant could be beneficial for the development of PA6 textile materials with multifunctional properties and the low ecotoxicity profile, while the PHED’s leaching and cytotoxicity limit their application involving the washing processes and direct contact with the skin.

## 1. Introduction

Flame retardants (FRs) represent a class of functional materials with different chemistries tailored for application to specific flammable materials, e.g., polymers or plastics, textiles, and foams. FR can be applied to the surface of combustible material or incorporated into the polymer matrix, either as a chemically bonded or as an additive, the main purpose being to impart a heat-activated chemical reaction that prevents sustained ignition or slows the spread of flame at the specific temperature [1,2]. However, many of the bulk and surface-incorporated flame retardants, as well as other additives, that are used to improve the properties of plastic and textile products are leached out during their lifetime (through washing, migration into air, atmospheric washout by precipitation), in the recycling phase, and during final disposal, ending up in the environment [3,4,5,6,7,8]. The health and environmental concerns related to the persistent, bioaccumulative and toxic (PBT) halogenated flame retardants stimulated the search for alternative solutions and the development of more sustainable flame retardants [1,2,9,10,11]. Organophosphorus compounds (OP), i.e., organophosphates, organophosphonates, organophosphinates, organoposphine oxide, and organophosphites, are currently being intensively investigated as alternatives to PBT flame retardants due to due the chemical versatility of phosphorus’ different oxidation states and their ability to provide effective flame retardant protection in both the condensed and gas phases [12,13,14,15,16,17,18,19,20].

Among the organophosphorus compounds, organophosphate esters are increasingly used in the flame retardant industry. However, organophosphate esters from both flame retardant and pesticide application are also ubiquitous in abiotic, including air, water, dust, soil, sediment, and sludge, and biotic matrices, including birds, fish, and human tissue [21,22,23]. The determined levels and their distribution in different biotic and abiotic matrices indicate different patterns due to different consumption levels, different bioaccumulation and biomagnification capacities, different metabolic pathways, etc. For example, Hou et al. reported that halogenated organophosphate FRs were the main pollutants in the river water samples studied, while non-halogenated organophosphate FRs were the dominant organophosphorus FRs in the sediments [24]. Estill et al. reported that concentrations of organophosphate FR in air in the manufacturing, construction, and service sectors were higher and more prevalent than those of polybrominated diphenyl ethers [25]. More detailed information about the concentrations of organophosphorus-based flame retardants in the abiotic and biotic matrices can be found in the literature [7,21,22,26,27]. Results from toxicity testing, epidemiological studies, and risk assessments indicate that health concerns exist at current exposure levels for both halogenated and non-halogenated organophosphate ester flame retardants [28]. In comparison to organophosphates that can be highly toxic to nontarget species including humans, phosphinates are considered to be less toxic than phosphates and phosphonates [29].

Accordingly, the emerging 9,10-dihydro-9-oxa-10-phosphaphenanthrene-10-oxide (DOPO) derivatives represent promising halogen-free flame retardant alternatives that have already found application in various polymers such as epoxy resins, polyurethane foams, engineering plastics, and polyester and polyamide fibers due to the highly efficient and versatile flame retardant activity [2,11,30,31,32,33]. Although flame retardant DOPO derivatives have found widespread application in the last decade, their toxicity profiles and bioaccumulation potentials as well as the toxicity profiles of the final flame retardant polymeric material have rarely been reported [34].

The firstly developed DOPO derivatives in 1972 were synthesized as the new organophosphorus compounds aimed to be used not only as flame retardants but also as insecticides and fungicides [35]. Despite this, the biological activity of flame retardant DOPO derivatives incorporated into polymeric materials has not been considered yet, although it could be highly beneficial for the development of multifunctional, flame retardant, antibacterial, and antifungal properties for textile materials. Their development is of high importance for modern society, as these multifunctional textile materials are designed to provide protection from various hazards, such as fire and heat, extreme environment conditions, and microbial pathogens [36,37]. As a result of the large surface area of textile materials, there is a high risk of combustion when they come into contact with flames and ignite, as well as the attachment and growth of microbial pathogens and their transmission [38,39]. The antibacterial and antifungal properties are highly valuable functionalities, not only in terms of healthcare and hygiene concerns but also to increase the longevity of materials, while the antipathogenic performance must be non-toxic to humans and the environment [40,41,42]. In addition to the transmission of pathogens, which pose a serious threat to health, the growth of microorganisms leads to fabric deterioration, malodors, and discoloration [43,44,45,46].

In our previous work, we have demonstrated the development of novel halogen-free flame retardant PA6 filament yarns with a highly efficient self-extinguishing properties achieved by the incorporation of 6′-(1-phenylethane-1,2-diyl)bis(dibenzo[c,e][1,2]-oxaphosphinine-6-oxide) (PHED) in the PA6 matrix [31]. To gain more insight into the functional properties of this flame retardant PA6 textile material and its toxicity profile, the present study focuses on the evaluation of the antibacterial and antifungal properties, cytotoxicity, leaching, and the leachate aquatic ecotoxicity. The flame retardant PA6 textile filaments were prepared using the in situ incorporation of nonreactive organophosphinate-bridged DOPO derivatives during the polymerization of ε-caprolactam, which was followed by the melt-spinning process. The antibacterial properties of the flame retardant PA6 fiber were tested against the Gram-negative bacterium Escherichia coli and the Gram-positive bacterium Staphylococcus aureus, whilst their antifungal properties were tested against the filamentous fungus Aspergillus niger and opportunistically pathogenic yeast Candida albicans. The in vitro cytotoxicity of the flame retardant PA6 fiber was determined and qualitatively evaluated for human primary mesenchymal stromal (stem) cell culture from adipose tissue (ADSCs) in a direct contact. The leaching behavior of the organophosphinate PHED flame retardant incorporated into the PA6 textile fibers and its influence on the aquatic ecotoxicity were evaluated using the duckweed Lemna minor.

## 2. Materials and Methods

### 2.1. Materials

**Figure 1:**
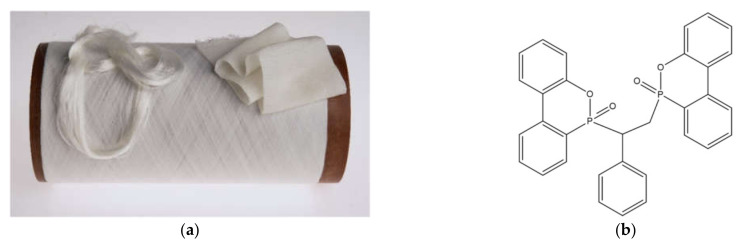
The flame-retardant (PA6)/bridged 9,10-dihydro-9-oxa-10-phosphaphenanthrene-10-oxide (DOPO) derivative nanocomposite filament yarns (**a**) and molecular structure of the flame-retardant bridged DOPO derivative (6′-(1-phenylethane-1,2-diyl)bis(dibenzo[c,e][1,2]-oxaphosphinine-6-oxide), PHED) (**b**).

The reference polyamide 6 (PA6) and the flame retardant polyamide 6 (PA6)/bridged DOPO derivative nanocomposite textile filament yarns used in this work were produced by our research group, as previously reported in Vasiljević et al. [31]. Briefly, in situ water-catalyzed ring-opening polymerization of ε-caprolactam was conducted in the presence of the flame retardant 6,6′-(1-phenylethane-1,2-diyl)bis(dibenzo[c,e][1,2]-oxaphosphinine-6-oxide) (PHED), followed by melt-spinning of nanocomposite filament yarns. The flame retardant PHED was synthesized in our laboratories according to the literature [47], and it was applied at concentrations of 10 and 15 wt %. The flame retardant PHED was applied at these two concentrations because we have previously shown that 10 wt % of this flame retardant additive provides the increased thermo-oxidative stability of the PA6 textile filament, while 15 wt % of this FR is required to achieve a substantial reduction in the flammability of the melt drops produced in the vertical flammability test of the fiber strand sample [31]. Therefore, depending on the potential application of the PA6/PHED textile filament, both concentrations used may be relevant. The corresponding samples were coded as PA6/10PHED and PA6/15PHED, respectively. For comparison, the PA6 reference sample was synthesized according to the same method in the absence of PHED. Figure 1 shows the flame retardant PA6/PHED nanocomposite filament yarns and molecular structure of the flame retardant PHED. The diameters of the PA6, PA6/10PHED, and PA6/15PHED were equal to 65, 69, and 81 μm, respectively. The calculated concentrations of phosphorus in the PA6 samples, which contained 10 and 15 wt % of the incorporated flame retardant PHED, respectively, corresponded to 1.16 and 1.74 wt %.

### 2.2. Antibacterial Properties

The antibacterial properties of the PA6, PA6/10PHED, and PA6/15PHED fiber samples were estimated for the Gram-negative bacterium *Escherichia coli (E. coli)* ATCC 11229 and the Gram-positive bacterium *Staphylococcus aureus* (*S. aureus*) ATCC 25923, according to the ASTM E 2149–01 standard method. The nutrient broth medium and the nutrient agar used for the antibacterial tests were obtained from Oxoid, CM0067, Basingstoke, UK and Biolife, 4018102, Milano, Italy, respectively. The inoculum, consisting of 10^5^ colony-forming units (CFU), was prepared for each bacterium. The inoculum was added into Milli-Q water in which the samples were immersed, so that the final concentration of bacteria corresponded to 10^4^ CFU. Each 1 g fiber sample was immersed in 20 mL of a nutrient broth medium of known bacterial concentration in a flask. After immersion, 40 μL of the inoculum was spread on nutrient agar plates, and the infused agar plates were incubated at 37 ± 2 °C for 24 h. These samples were labeled “time of immersion 0” and were used as the basis for the bacterial reduction calculation. The rest of the inoculum with the immersed sample was shaken using a wrist-action shaker for 2 h at 180 rpm, which was followed by the spreading of 40 μL of the inoculum on agar plates and the incubation of the plates at 37 ± 2 °C for 24 h. After incubation, the bacterial colonies grown on the agar plates were counted, and the bacterial reduction was calculated. For each fiber sample, two parallel assessments were performed. The reduction in the number of bacteria, *R_bacteria_*, was calculated as follows:

(1) $$R_{bacteria}=\frac{\left(B-A\right)}{B}\;\times 100\;\%$$

where *A* is the number of bacteria in a flask after 2 h of contact time with the sample, and *B* is the number of bacteria in a flask containing a sample before shaking. The *R_bacteria_* value is reported as the mean value with the standard error.

### 2.3. Antifungal Properties

The antifungal properties of the PA6, PA6/10PHED, and PA6/15PHED yarn samples were estimated for the filamentous reference fungus *Aspergillus niger* (*A. niger*) and reference yeast *Candida albicans* (*C. albicans*) (Figure S1), as determined according to the standard ISO 13629-2:2014(E) with modifications described below. The fungal strains used in the present study, *A. niger* (EXF-311) and *C. albicans* (EXF-525), were purchased from the Ex Culture Collection (Infrastructural Centre Mycosmo, Department of Biology, Biotechnical Faculty, University of Ljubljana, Ljubljana, Slovenia). The cultures were maintained on nutrient medium Sabouraud dextrose agar and kept at 4 °C until the experiments were performed.

The antifungal activity was determined by the plate count method using the untreated PA6 fibers as the control sample. The inoculum suspension was prepared by resuspending the spores of *A. niger* and the yeast cells of *C. albicans* in sterile water with 0.05% of detergent (dioctyl sodium sulfosuccinate) and counting the cell number in a hemocytometer; then, they were diluted 10 times in 5% Sabouraud dextrose broth to obtain the final concentration of 2.5 × 10^5^ spores or cells mL^−1^. PA6, PA6/10PHED, and PA6/15PHED filament samples were cut into 0.5 cm long fibers, which were afterwards sterilized for 15 min at 121 °C and 1.2 atm. Each fiber sample was prepared in eight replicates and inoculated with 0.2 mL spore suspension according to the absorption method. Four samples were processed immediately (0 h), while the other four were incubated at 30 °C for 48 h. Before and after 48 h of incubation, neutralization solution according to ISO 13629-2:2014(E) was added to each inoculated sample, shaken vigorously, and serially diluted in 0.05% detergent solution. Each of the four replicates of the samples was serially diluted to 0.001, and 0.1 mL was aseptically spread on the surface of Sabouraud dextrose agar in triplicates. After their growth on agar medium (30 °C for 48 h), the colony-forming units were counted visually. The fungal concentration in the solution was calculated as follows:

(2) $$N=\frac{\sum C}{V\times 1.1\times d}$$

where *N* is the fungal concentration in CFU/mL, Σ*C* is the sum of the CFU counted on two dishes retained from two successive dilutions, *d* is the first dilution step above two successive dilutions, 1.1 is a coefficient to combine the two successive dilutions from ISO 13629-2:2014(E), and *V* is the volume of inoculum placed in each dish. The reduction in the number of grown colonies, *R_f_*, was calculated as follows:

(3) $$R_{fungus}=\frac{\left(N_{0}-N_{48}\right)}{N_{0}}\;\times 100\;\%$$

where *N*_0_ is the fungal concentration in the inoculum suspension (colony-forming units per millimeter) prior to incubation with fibers, and *N*_48_ is the fungal concentration in inoculum suspension after 48 h of incubation with fibers. The *R*_fungus_ value is reported as the mean value with standard deviation.

### 2.4. Cytotoxicity

The in vitro cytotoxicity of the PA6 and PA6/15PHED fiber samples was determined and qualitatively evaluated for human primary mesenchymal stromal (stem) cell culture from adipose tissue (ADSCs) in a direct contact test system according to the standard ISO 10993-5:2009. The negative (produces no cytotoxic response) and positive (produces a cytotoxic response) controls were used in accordance with the standard ISO 10993-12:2012. The PA6 and PA6/15PHED fiber samples were fixed to the bottom of the cell culture vessel, and the primary human ADSCs were seeded uniformly into the cell culture medium throughout the cell culture vessel (including the sample surface). The samples with cells were incubated for 7 days at 37 °C, 5% CO_2_. The morphology of the cells in the vicinity of the samples was examined microscopically. After 7 days incubation, the cells were also examined after using the cytochemical staining 3-(4,5-dimethylthiazol-2-yl)-2,5-diphenyltetrazolium bromide (MTT). Prior to the examination, the samples were sterilized in an autoclave.

### 2.5. Leaching Properties

To evaluate the leaching of the PHED compound from PA6 fibers into freshwaters, 0.1 g of each tested fiber sample was placed into Erlenmeyer flasks and dispersed in 100 mL of Steinberg medium (ISO 20079:2005). The Erlenmeyer flasks were placed on an orbital shaker and incubated under 150 rpm for three weeks at 22 ± 2 °C. The three-week leaching in Steinberg medium (pH = 5.8) simulated an intensive exposure of the fibers to rainwater (typically pH = 5–6). The concentration of the fibers in Steinberg medium was 1 g/L (solid-to-volume ratio equal to 1/1000). Although the leaching properties of many solids (e.g., soils) are often assessed at lower solid-to-volume ratios (e.g., up to 1/25, OECD Test No. 106, 2000), the simulation of leaching properties of artificial surfaces often requires higher solid-to-volume ratios to ensure relevant environmental exposure (e.g., 1.25/1000 in Soroldoni et al. [48]). After incubation, the fiber samples were carefully removed from each Erlenmeyer flask, dried, and stored at room temperature in closed jars. The medium was stored in a freezer at −20 °C.

Additionally, the PA6 textile filament samples were washed in an AATCC Atlas Launder-O-Meter standard device (SDL Atlas, Rock Hill, SC, USA). One washing cycle in a Launder-O-Meter (ISO 105-C06:2010 standard method) with ten steel balls provides an accelerated washing treatment equivalent to 5 domestic washes. The washing of the filament samples was carried out in a solution of standard detergent (SDC ECE Phosphate reference detergent (B) (SDC Type 3, SDC Enterprises Limited, Holmfirth, UK) at a concentration of 4 g/L at 40 °C for 45 min. After washing, the samples were rinsed twice in distilled water at 40 °C for 1 min, then rinsed in tap water, and finally dried in air at room temperature.

#### 2.5.1. Fibers Properties after Leaching and Washing

Scanning electron microscopy (SEM) was performed on a Zeiss SUPRA 35VP SEM microscope (Jena, Germany). The leached samples were coated with Cr.

The changes in the phosphorus content of the textile fibers prior to and after leaching as well as prior and after washing were investigated by means of laser ablation elemental mass spectrometry (LA-ICP-MS). Bundles of fibers were mounted to microscope glass slides and placed into the ablation cell of the LA instrument (Teledyne Photon Machines, Bozeman, MT, USA, Analyte G2, a nanosecond ArF* excimer instrument, equipped with HelEx2 dual volume ablation cell). A laser beam (80 µm, square shape, 20 Hz repetition rate) was traversed over the fiber bundle (4 mm in length), and ablated aerosol, consisting of ablated fiber particles with an equal stoichiometry as the fibers, was transported by an He carrier gas stream into the ICP-MS instrument (Agilent Technologies 7900x ICP-MS, Tokyo, Japan) where the signal intensity for 31P was recorded. The intensities of the 31P signal, which provided the information on the content of phosphorus, were measured relative to neat PA6 fibers.

#### 2.5.2. Ecotoxicity of Leachates

For evaluation of the ecotoxicity of leachates from PA6, PA6/10PHED, and PA6/15PHED fibers, the duckweed *Lemna minor* was used (Figure S2). It was cultivated in Steinberg medium (ISO 20079:2005) under controlled conditions. Toxicity testing was performed in 6-well culture plates (TPP^®^, Switzerland), and each well was filled with 10 mL of undiluted leachate corresponding to the PA6, PA6/10PHED, and PA6/15PHED samples. The control tests contained 10 mL of Steinberg medium. Each experiment included four replicates of the same leachate, and each experiment was repeated twice. The initial number of fronds was six, leaving more than 50% of the surface area for further growth. The toxicity test was performed in a climate test chamber at 24 ± 2 °C and high humidity (>70%) to minimize the evaporation of the test media. All treatments were illuminated by daylight fluorescent lamps with a photoperiod of 16/8 h (light/dark) at a light intensity of ≈4000 lx at the plant level [49].

The experiment proceeded for seven days, and at the end of the experiment, the number of fronds was counted. The average specific growth rate was calculated according to (ISO 20079:2005) as follows:

(4) $$\mu =\frac{ln\left(N_{j}\right)-ln\left(N_{i}\right)}{t}$$

where *µ* (1/d) is the average specific growth rate, *Nj* (/) is the number of fronds at the end of the experiment, *Ni* (/) is the number of fronds at the beginning of the experiment, and *t* (d) is the time period of exposure (7 days).

## 3. Results and Discussion

As a chemically recyclable textile on the industrial level, PA6 textile materials are important for the textile industry and society from the sustainability point of view. In our previous work [31,50], we have developed a method for upgrading the inherent properties of this fiber in terms of its flame retardancy and increased thermal stability. The method of production of the flame retardant PA6 fibers with homogeneously distributed, nanodispersed, bridged DOPO derivatives includes its physical incorporation into the polymer matrix. Therefore, assessment of the leaching ability and potential biological activity of the incorporated PHED is of high importance for the future application of this flame retardant PA6 fiber.

### 3.1. Antibacterial and Antifungal Properties

In order to investigate the antibacterial activity of the incorporated PHED compound in the PA6 textile fibers, the pathogenic bacteria *E. coli* and *S. aureus* were used as models for Gram-negative and Gram-positive bacteria, respectively [51,52]. For the evaluation of the antifungal activity, two morphologically and physiologically different fungi were selected: firstly, the cosmopolitan, filamentous fungus *A. niger*, which is a common cause of food decay and extensive biodeterioration of untreated textiles; and secondly, the yeast *C. albicans*, which is a component of the human microbiome colonizing the vaginal and gastrointestinal tracts. *C. albicans* can cause several health conditions from mild to some of the most threatening human mycotic infections [39,52,53], whereas *A. niger* is generally considered to be safe.

**Figure 2:**
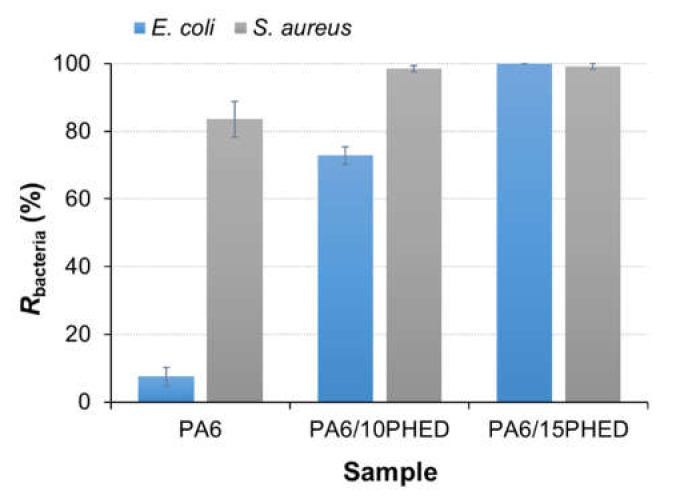
Bacterial reduction, *R*_bacteria_, of *E. coli* and *S. aureus* obtained for the inoculums that were in contact with the PA6, PA6/10PHED, and PA6/15PHED fiber samples. The error bars represent the standard error of the mean. PA6: polyamide 6, PA6/10PHED and PA6/15PHED: PHED in the PA6 matrix during the in situ polymerization process at concentrations equal to 10 and 15 wt %, respectively.

**Figure 3:**
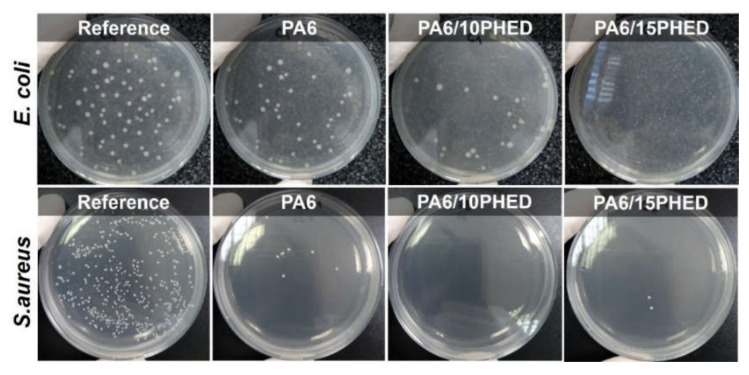
The grown *E. coli* and *S. aureus* bacteria on the agar plates after incubation, obtained for the reference inoculum and inoculums that were in contact with the PA6, PA6/10PHED, and PA6/15PHED fiber samples.

The results for the reduction in *E. coli* and *S. aureus* growth achieved in the presence of PA6, PA6/10PHED, and PA6/15PHED fiber samples are presented in Figure 2 and Figure 3. The results indicate the different abilities of the PA6, PA6/10PHED, and PA6/15PHED samples to influence bacterial growth in the inoculum. Starting with the PA6 sample, the reduction in *S. aureus* growth in inoculum reached 84%, whilst the reduction in *E. coli* was equal to 7.5%. The reason for this lies in the different abilities of the tested bacteria to adhere to the PA6 fiber surface. Compared to *E. coli*, the adherence of *S. aureus* on the textile fibers under stationary or agitated suspensions is stronger because this bacteria possess intercellular adhesins, which are used as adhesion-promoting materials [51,54,55], whilst the *E. coli* adhesion is mainly driven by an interplay of van der Waals, electrostatic, and hydrophobic interactions [56]. In the case of the PA6/10PHED and PA6/15PHED, the antibacterial activity of the incorporated PHED reduced the growth of the both *E. coli* and *S. Aureus*. An excellent antibacterial activity, with 100% and 99% reduction in *E. coli* and *S. Aureus* growth, respectively, was achieved in the case of PA6/15PHED, indicating PHED’s bactericidal effect.

**Figure 4:**
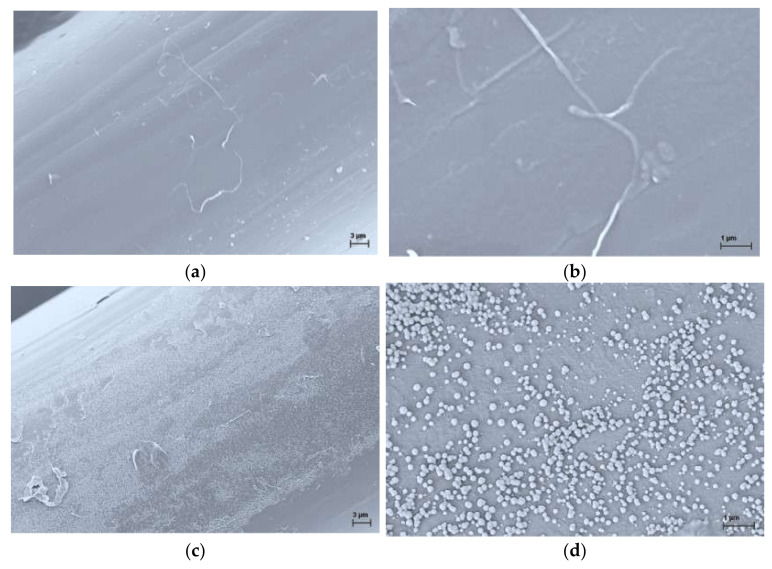

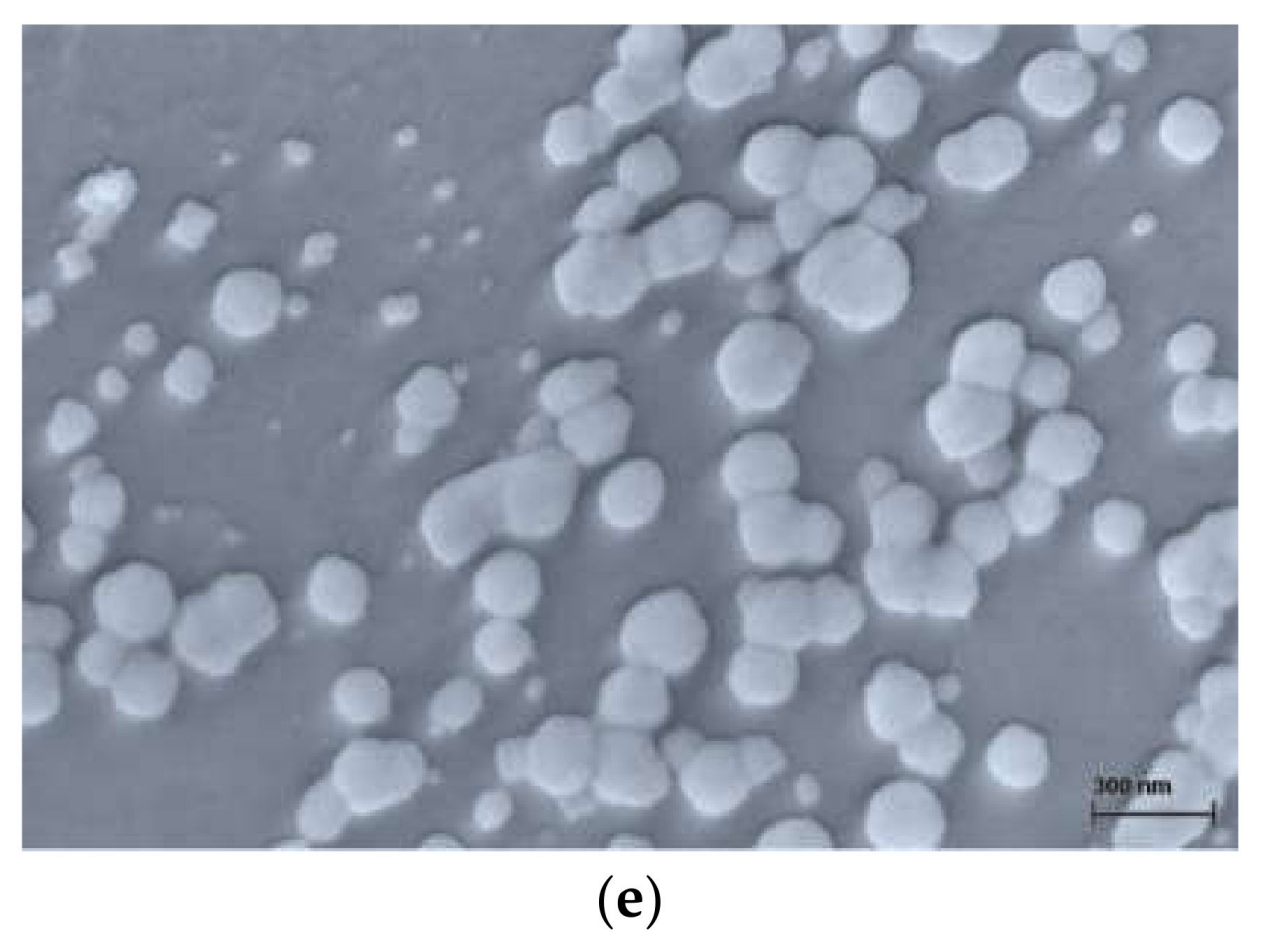
Representative SEM images of the surface morphology of the PA6 fibers after incubation of *E. coli* ((**a**,**b**) at magnification factor/working distance of 5000×/4.0 mm and 25,000×/4.0 mm, respectively) and *S. Aureus*, ((**c**–**e**) at magnification factor/working distance of 5000×/4.0 mm, 25,000×/4.1 mm and 100,000×/4.1 mm, respectively).

**Figure 5:**
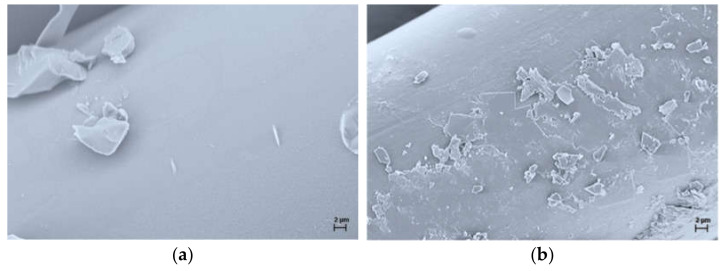
Representative SEM images of the surface morphology of the PA6/15PHED fibers after incubation of *E. coli* ((**a**) at magnification factor/working distance of 5000×/4.2 mm) and *S. Aureus*, ((**b**) at magnification factor/working distance of 5000×/4.1 mm).

The surface morphology of the PA6 samples after incubation of *E. coli* and *S. Aureus* was observed with a scanning electron microscope, and the representative SEM images are shown in Figure 4. These results showed that *S. Aureus*, in contrast to *E. coli*, was abundantly adsorbed on the PA6 fiber surface, and the bacteria were observed as single cells and cell cluster, confirming the suitability of the PA6 fiber surface for the growth of *S. Aureus*. According to the SEM analysis of the surface morphology of the PA6/15PHED fibers after incubation of *E. coli* and *S. Aureus* (Figure 5), the adhered bacteria could not be observed, which confirmed the bactericidal effect of PHED.

According to the results for the reduction in growth of *E. coli* and *S. aureus* obtained in the presence of the PA6/10PHED fiber sample (Figure 2), it can be noted that the Gram-positive bacterium *S. aureus* is more sensitive to PHED than the Gram-negative bacterium *E. coli*. Therefore, it can be assumed that the antimicrobial effect of PHED on the growth of Gram-negative *E. coli* and Gram-positive *S. aureus* is different due to the different cell wall structures of the bacteria used. Namely, the cell wall of Gram-positive bacterium consists of a 20–80 nm thick peptidoglycan layer, whereas the cell wall of Gram-negative bacteria consists of an outer membrane and a thinner (<10 nm) peptidoglycan layer. Due to the different composition of the outer shells, the investigated bacterial species react differently to external stress, e.g., antimicrobial agents [57]. Although the mechanism of PHED’s antimicrobial action has not been reported in the literature, it is reported that the antibacterial activity of synthetic organophosphonate and organophosphinate compounds can be attributed to their competing action with biosynthesized analogs (i.e., carboxylic acids and phosphate esters naturally occurring in living organisms) for binding to the active sites of the enzymes, thereby acting as potent inhibitors [58,59]. Therefore, it can be assumed that the organophosphinate PHED could chemically bind to the enzyme’s active sites in the bacteria cells where it hinders or blocks the catalytic activity of the enzyme in the reactions vital for cell growth.

**Figure 6:**
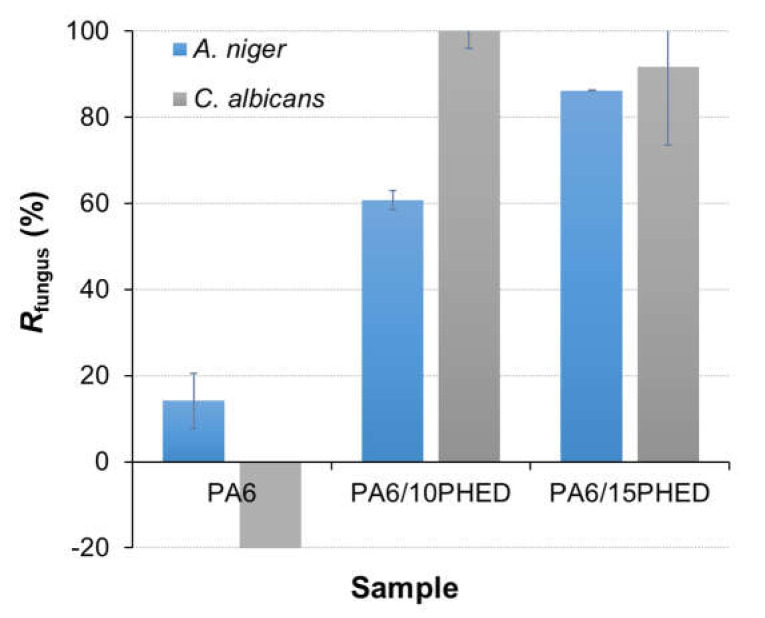
Fungal reduction, *R*_fungus_, in *A. niger* and *C. albicans* achieved in the presence of PA6, PA6/10PHED, and PA6/15PHED fiber samples. The error bars represent the standard error of the mean.

For the assessment of the antifungal properties of the fibers, two morphologically completely different fungal species were selected, namely *A. niger* and *C. albicans*. *A. niger* is a filamentous fungus, and its spores cannot multiply by cell division; they can either die or survive on the fiber surface. On the other hand, the yeast *C. albicans* budding cells could either die, stop dividing/budding, or continue to multiply on the fiber surface. The antifungal effect to both tested fungal species was measured as the reduction of their growth in the presence of the PA6, PA6/10PHED, and PA6/15PHED fiber samples. The results for the reduction in *A. niger* and *C. albicans* growth achieved in the presence of the PA6, PA6/10PHED, and PA6/15PHED fiber samples are presented Figure 6. The results obtained for the *A. niger* show that incorporated PHED increased the antifungal activity of PA6 fibers, inducing the inhibition of *A. niger* spore germination from 14.2% for PA6 to 60.8% and 86.2% for PA6/10PHED and PA6/15PHED samples, respectively. The intensified inhibition of the germination of *A. niger* spores with the increased PHED concentration in the fibers confirmed the PHED’s fungicidal action. Furthermore, the PHED’s fungicidal action was even enhanced in the case of the yeast *C. albicans*, where almost no colonies of *C. albicans* grew when PHED was present in the sample (a 100% and 91.8% reduction in yeast cells for PA6/10PHED and PA6/15PHED, respectively). In contrast to *A. niger*, where the number of spores on the PA6 fibers was reduced by 14.2% after 48 h, for *C. albicans*, the yeast cells multiplied on the PA6 fibers, their number reaching more than 832.4% of the initial number prior to incubation. This suggests that PA6 fibers without PHED do not affect the multiplication of fungal cells nor do they have a weak fungicidal effect as was suggested based solely on the results of *A. niger*.

The modest reduction in the germination of *A. niger* spores in the presence of the PA6 sample indicates that some of the spores did not survive on the fibers or remained adsorbed in the fibers. However, this phenomenon was not observed for *C. albicans*, which was possibly because these cells multiplied during incubation or because of the different mechanisms of adherence. Whilst the investigation of the mechanism of PHED’s antifungal activity against the *A. niger* and *C. albicans* bacteria is beyond the scope of this work, it can be noted that the fungicidal action of organophosphorus compounds induces disturbances in the fungi’s metabolism [60], and it induces membrane dysfunction by inhibiting the formation of phosphatidylcholine, thus causing an imbalance in membrane phospholipids [61,62]; it can also limit cell growth by inhibiting the chitin synthase enzyme [63].

**Figure 7:**
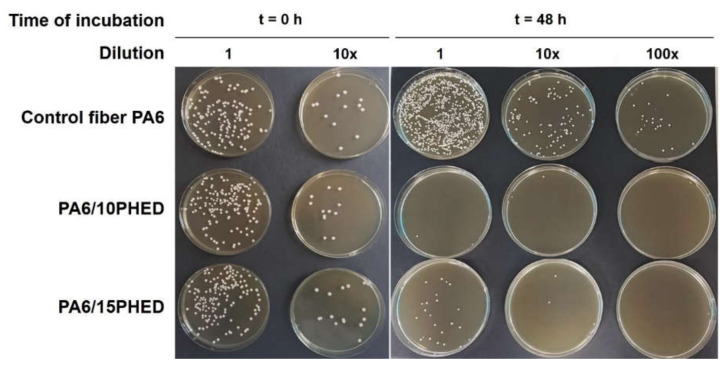
Growth of *C. albicans* after transferring the undiluted, 10× diluted, and 100× diluted inoculated samples prior to and after 48 h of incubation.

To support the above results and to illustrate the outcome of the test performed, Figure 7 shows photographs of the colonies belonging to *C. albicans* after transfer of the undiluted, 10-fold diluted, and 100-old diluted inoculated samples prior to and after 48 h of incubation. The photographs for *A. niger* are not shown here, because they would provide the same information as those for *C. albicans*. Additionally, the colonies of *A. niger* on agar plates were much less visible to the naked eye in comparison to the *C. albicans.* The latter shows a more representative photogenic growth due to filamentous growth with gentle hyphe spreading from the spore. These results demonstrate the almost complete inhibition of growth of the colonies of the yeast *C. albicans* in the presence of the PA6/10PHED and PA6/15PHED samples, confirming the excellent fungicidal effect of the organohosphinate PHED.

### 3.2. Cytotoxicity Properties

**Figure 8:**
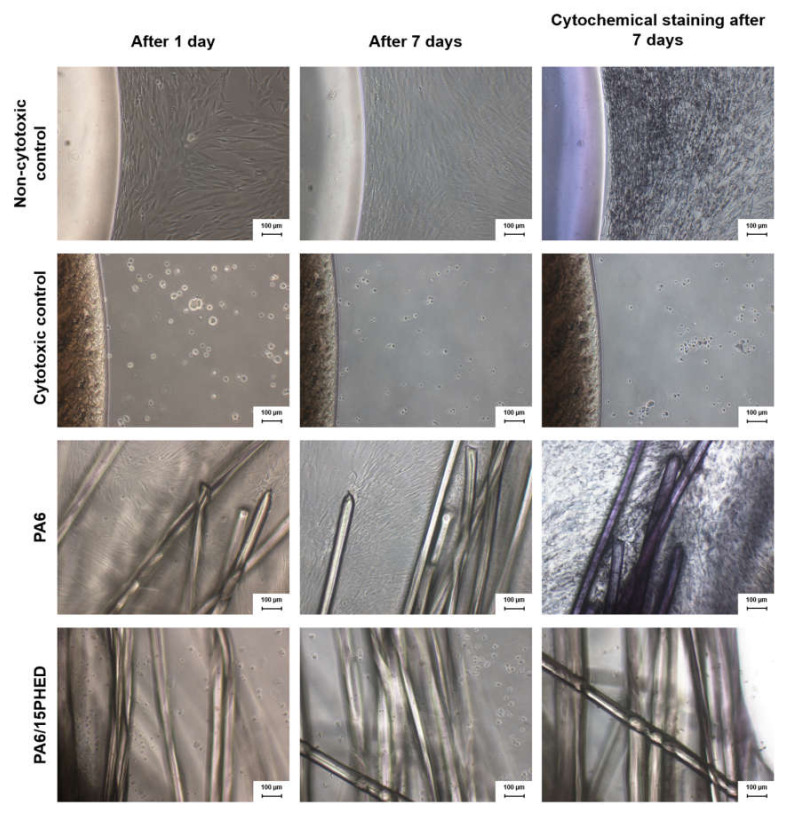
The results of a daily monitoring of the grown human stromal (stem) cell culture from adipose tissue (ADSCs) in the negative (non-cytotoxic) and positive (cytotoxic) controls, and in the immediate vicinity of the PA6 and PA6/15PHED fiber samples after 1 and 7 days of incubation as well as after cytochemical staining of the 7-day incubated samples.

**Figure 9:**
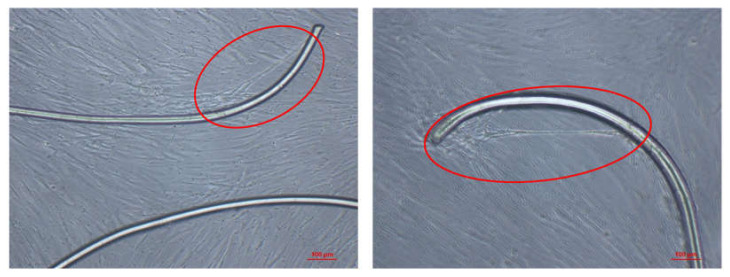
The images of a human cells attached to the PA6 fiber.

The cytotoxicity of the PA6 and PA6/15PHED samples was evaluated to investigate their toxic effect on human cells during prolonged and direct contact with these materials. To the best of our knowledge, the cytotoxicity of flame retardant textile materials has rarely been reported in the literature, although the use of organophosphorus flame retardants in textile materials is common. The results of daily monitoring of a human primary mesenchymal stromal (stem) cell (MSC) culture in the negative (non-cytotoxic) and positive (cytotoxic) controls and in the immediate vicinity of PA6 and PA6/15PHED after 1 and 7 days are presented in Figure 8. The metabolically active human cells in contact with the PA6 sample exhibited no cytotoxic effect as the cells grow undisturbedly next to the fibers, which is marked with a red circle in Figure 9. Moreover, the cells showed the affinity to attach to and grow on the PA6 fibers in vitro. However, in the case of the PA6/15PHED sample, complete growth inhibition was observed after the first incubation day, which indicates that this flame retardant could be toxic to the growth and viability of human cells when a human tissue comes into prolonged contact with this textile material.

Figure 8 also shows that the use of the cytochemical staining MTT in the case of the PA6 sample (and the negative control) highlighted the high number of grown cells (in violet color), whereas in the case of the PA6/15PHED sample (and the negative control), no visible viable cells were present after 7-day incubation. Therefore, at the applied concentration of PHED required for effective flame retardant protection, the flame retardant PHED compound induces the cytotoxic properties of the PA6/15PHED textile material. The implication can be made that this flame retardant textile material would be suitable for applications where the fabric is not in direct contact with human skin.

### 3.3. Leaching Properties

**Figure 10:**
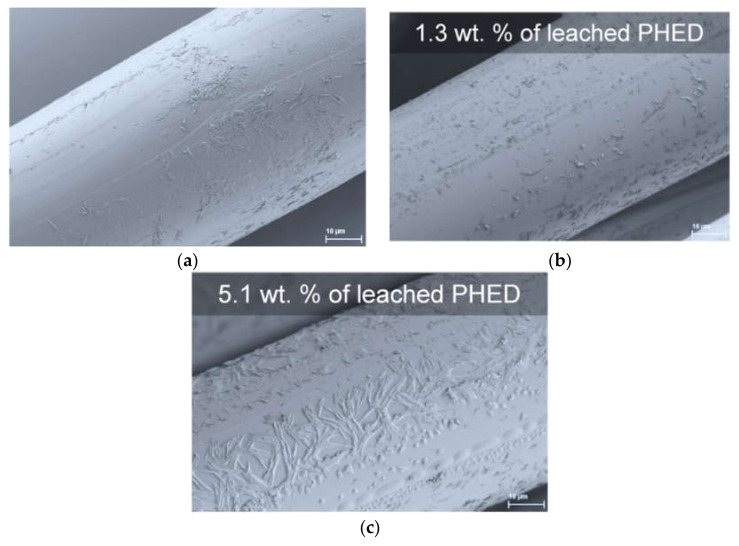
Representative SEM images of the surface morphology of the leached (**a**) PA6, (**b**) PA6/10PHED, and (**c**) PA6/15PHED fibers.

The leaching properties of the PHED compound incorporated into the PA6 textile fiber were tested for the fibers after one washing cycle, which is according to the standard equal to five domestic washings. Additionally, the leaching properties were tested after exposing the samples to synthetic freshwater for a period of three weeks, which simulates exposure to an intensive rain event. Afterwards, the fibers were analyzed by LA-ICP-MS for the content of phosphorus present in unleached PA6, PA6/10PHED, and PA6/15PHED fibers as well as the leached ones. The results showed that the phosphorus content in the washed PA6/15PHED fibers decreased by 12%, whilst in the leached PA6/10PHED and PA6/15PHED, fibers decreased by 13% and 34%, respectively. These results indicate that organophosphinate PHED physically incorporated into the PA6 fibers tends to leach out, which is a common phenomenon for the additives physically incorporated into the polymer matrix. The more pronounced leaching in the case of the fibers exposed to synthetic freshwater for a three-week period could be assigned to prolonged contact with water medium. Furthermore, the leached fibers were examined by SEM, and the representative images of the surfaces of the leached PA6, PA6/10PHED, and PA6/15PHED fibers are shown in Figure 10.

These results reveal that after the leaching process, heterogeneities had appeared in the originally relatively smooth surfaces of the PA6, PA6/10PHED, and PA6/15PHED fibers [31], which was probably caused not only by the leaching of the PHED compound, but also by the PA6 oligomers. These appear on the fiber surface presumably because of the hydrophobicity of both the PHED and PA6 oligomers. For the same reason, white-colored films were observed to be adsorbed on the glass walls of the Erlenmeyer flasks in the case of all three samples.

**Figure 11:**
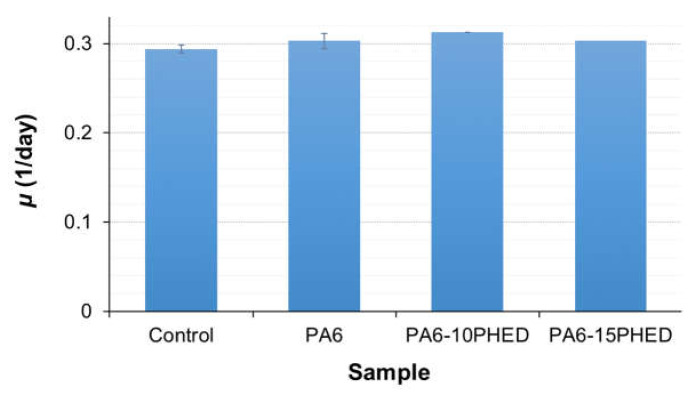
The average specific growth rate of the *L. minor* grown in the control and PA6, PA6/10PHED, and PA6/15 PHD leachates. The error bars represent the standard error of the mean.

Subsequently, the collected leachates were submitted to the ecotoxicity test with duckweed *L. minor*. According to the phosphorus content determined for the fiber samples by LA-ICP-MS after seven-day incubation in Steinberg medium, the concentrations of leached PHED were calculated to be 1.3 × 10^−3^ g/L and 7.65 × 10^−3^ g/L for the PA6/10PHED and PA6/15PHED fibers, respectively. The results for the ecotoxicity test after seven days of incubation are presented in Figure 11. The leachates from the PA6, PA6/10PHED, and PA6/15PHED fiber samples did not negatively affect the specific growth rate of the duckweed in comparison to the control sample. No structural or pigmental differences between the grown *L. minor* fronds were observed, confirming that the tested leachates did not affect the reproductive potential and growth of the duckweed. As a DOPO derivative, the PHED flame retardant can be considered to have a low ecotoxicity profile; e.g., Waaijers et al. demonstrated the low toxicity of DOPO to the freshwater crustacean *Daphnia magna* [64]. Similarly, Liu et al. tested the toxicity of a 10-(2,5-dihydroxyl phenyl)-9, 10-dihydro-9-oxa-10-phosphaphenanthrene-10-oxide (DOPO-HQ) and no effect was observed on three trophic levels: the algae *Pseudokirchneriella subcapitata*, the crustacean *Daphnia magna*, and the fish *Gobiocypris rarus* at the saturation water solubility [65]. It can be assumed that the low toxicity of PHED is caused by its low solubility in water and low bioavailability; as described above, the leached PHED was adsorbed onto the glass walls of the Erlenmeyer flasks, and thus was not bioavailable to *L. minor*. Similar behavior can be expected in the environment, i.e., PHED being insoluble in the water adsorbed in the mineral fractions of soils and sediments with a low bioavailability.

Therefore, it can be concluded that the antimicrobial activity of the incorporated PHED flame retardant endowed the PA6 fiber flame retardancy with antibacterial and antifungal properties, which was accompanied by the cytotoxicity. In addition, this textile material can be considered to have a low aquatic ecotoxicity profile with the respect to the applied tests.

## 4. Conclusions

Herein, the antibacterial, antifungal, leaching, and aquatic ecotoxicity properties of the novel flame retardant PA6/bridged DOPO derivative nanocomposite textile fibers were evaluated. The results revealed the targeted biological activity of this bridged DOPO derivative toward bacteria and fungi, while at the applied concentration of PHED, the PA6/10PHED, and PA6/15PHED fiber samples showed the cytotoxic properties. The water leachates from the PA6, PA6/10PHED, and PA6/15PHED fiber samples did not show any negative impact on the freshwater duckweed *L. minor*, which was probably due to the low bioavailability of the leached PHED to the plant, which was assigned to the low solubility and high hydrophobicity of the PHED compound. The obtained results demonstrate the importance of the applied methodology from the perspective of minimizing the risk for potential health and environmental impacts, where toxicology, safety, and circularity parameters should be considered as the foundations for building the design process of flame retardant systems. Additionally, targeted biological activity should be explored, as this property of organophosphorus flame retardants can be considered as highly beneficial for the development of textile materials with multifunctional protective properties. The leaching properties of the PHED physically incorporated into the PA6 textile fibers and its cytotoxicity demonstrate the feasibility of technical applications with this kind of textile, where the material is not exposed to intensive washing or possible rain events and is not in direct contact with the skin. In addition, leaching of the PHED could potentially be inhibited by applying a water repellent coating to the PA6/PHED textile filament, as the water repellent surface would minimize contact with water.

## Data Availability

Not applicable.

## Supplementary Materials

The following are available online at https://www.mdpi.com/2073-4360/13/6/905/s1, Figure S1: *A. niger* (upper left) and *C. albicans* (upper right) grown on agar medium at 30 °C for 7 and 5 days, respectively. Micrograph of *A. niger* conidia (lower left) and *C. albicans* cells (lower right) using light microscopy with differential interference contrast., Figure S2. The *Lemna minor* fronds at the beginning of the experiment.
