# Supplementary material for: New Insights into Antibacterial and Antifungal Properties, Cytotoxicity and Aquatic Ecotoxicity of Flame Retardant PA6/DOPO-Derivative Nanocomposite Textile Fibers
Source: Polymers (Basel). 2021 Mar 15;13(6):905. doi: 10.3390/polym13060905 (PMC7998799; doi:10.3390/polym13060905)
Supplement: Supplementary file 1 [file polymers-13-00905-s001.pdf]

Supporting Information

New Insights into Antibacterial and Antifungal Properties, Cytotoxicity, and Aquatic Ecotoxicity of Flame Retardant PA6/DOPO-Derivative Nanocomposite Textile Fibers

Jelena Vasiljević [1,*], Danaja Štular [2], Gabriela Kalčíková [3], Janja Zajc [4], Matic Šobak [2], Andrej Demšar [1], Brigita Tomšič [1], Barbara Simončič [1], Marija Čolović [2], Vid Simon Šelih [2] and Ivan Jerman [2]

[1] Faculty of Natural Sciences and Engineering, University of Ljubljana, Aškerčeva 12, 1000 Ljubljana, Slovenia; andrej.demsar@ntf.uni-lj.si (A.D.); brigita.tomsic@ntf.uni-lj.si (B.T.); barbara.simoncic@ntf.uni-lj.si (B.S.)
[2] National Institute of Chemistry, Hajdrihova 19, 1000 Ljubljana, Slovenia; danaja.stular@ki.si (D.S.); matic.sobak@ki.si (M.C.); marija.colovic@ki.si; vid.selih@ki.si (V.S.S.); ivan.jerman@ki.si (I.J.)
[3] Faculty of Chemistry and Chemical Technology, University of Ljubljana, Večna pot 113, 1000 Ljubljana, Slovenia; gabriela.kalcikova@fkkt.uni-lj.si
[4] Agricultural Institute of Slovenia, Hacquetova 17, 1000 Ljubljana, Slovenia, Slovenia; janja.zajc@kis.si
* Correspondence: jelena.vasiljevic@ntf.uni-lj.si; Tel.: +386 1 20 03 255

Citation: Vasiljević, J.; Štular, D.; Kalčíková, G.; Zajc, J.; Šobak, M.; Demšar, A.; Tomšič, B.; Simončič, B.; Čolović, M; V.S., Šelih; I. Jerman. New Insights into Antibacterial and Antifungal Properties, Cytotoxicity, and Aquatic Ecotoxicity of Flame Retardant PA6/DOPO-Derivative Nanocomposite Textile Fibers. *Polymers* **2021**, *13*, x. https://doi.org/10.3390/polym13060905

Academic Editor: Marco Zanetti

Received: 24 February 2021
Accepted: 12 March 2021
Published: date

Publisher's Note: MDPI stays neutral with regard to jurisdictional claims in published maps and institutional affiliations.

Copyright: © 2021 by the authors. Licensee MDPI, Basel, Switzerland. This article is an open access article distributed under the terms and conditions of the Creative Commons Attribution (CC BY) license (http://creativecommons.org/licenses/by/4.0/).

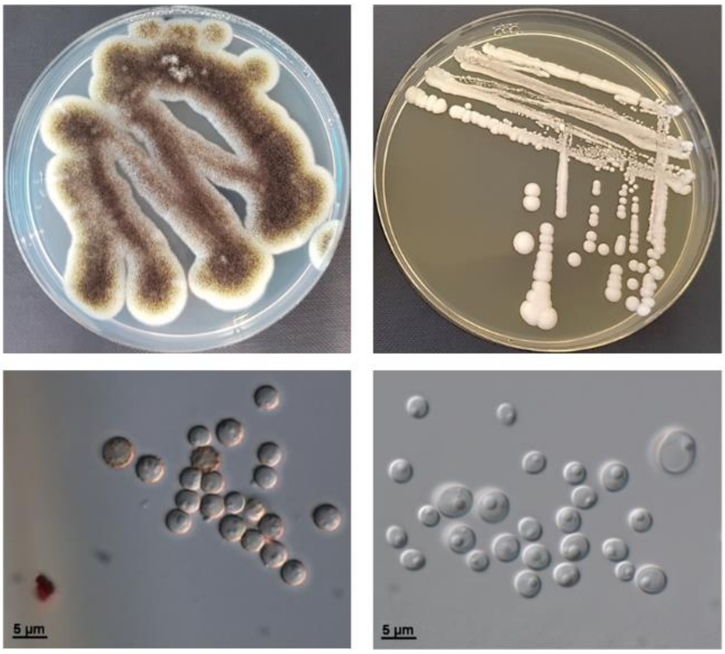

Figure S1. *A. niger* (upper left) and *C. albicans* (upper right) grown on agar medium at 30 °C for 7 and 5 days, respectively. Micrograph of *A. niger* conidia (lower left) and *C. albicans* cells (lower right) using light microscopy with differential interference contrast.

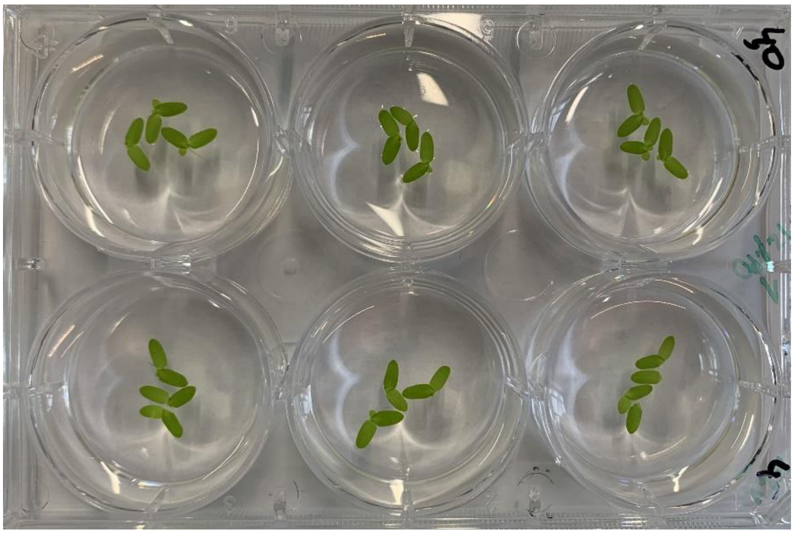

Figure S2. The *Lemna minor* fronds at the beginning of the experiment.
